# Cerebrolysin Use in Patients with Liver Damage—A Translational Study

**DOI:** 10.3390/life12111791

**Published:** 2022-11-04

**Authors:** Shandiz Morega, Andrei Gresita, Smaranda Ioana Mitran, Madalina Iuliana Musat, Ianis Kevyn Stefan Boboc, Victor Gheorman, Ion Udristoiu, Carmen Valeria Albu, Costin Teodor Streba, Bogdan Catalin, Ion Rogoveanu

**Affiliations:** 1 U.M.F. Doctoral School Craiova, University of Medicine and Pharmacy of Craiova, 200349 Craiova, Romania; 2 Experimental Research Centre for Normal and Pathological Aging, University of Medicine and Pharmacy of Craiova, 200349 Craiova, Romania; 3 Department of Biomedical Sciences, New York Institute of Technology, Old Westbury, NY 115680-8000, USA; 4 Department of Physiology, University of Medicine and Pharmacy of Craiova, 200349 Craiova, Romania; 5 Department of Psychiatry, University of Medicine and Pharmacy of Craiova, 200349 Craiova, Romania; 6 Department of Neurology, University of Medicine and Pharmacy of Craiova, 200349 Craiova, Romania; 7 Research Center of Gastroenterology and Hepatology, University of Medicine and Pharmacy of Craiova, 200349 Craiova, Romania; 8 Gastroenterology Department, University of Medicine and Pharmacy of Craiova, 200349 Craiova, Romania

**Keywords:** NAFLD, liver damage, anxiety, Cerebrolysin, neurogenesis

## Abstract

**Simple Summary:**

Given the prevalence of non-alcoholic fatty liver disease (NAFLD), there is a high chance that some of these patients can develop acute life-threatening events such as stroke. Our aim was to understand if the use of Cerebrolysin could be an option for stroke patients with altered liver enzymes levels. We also wanted to evaluate whether the treatment could reverse the inhibition of hippocampal neurogenesis in mice receiving methionine/clonidine deficiency (MCD) food. Cerebrolysin proved safe for clinical use in stroke patients with liver damage, although it could not reverse the inhibition of hippocampal neurogenesis to mice fed MCD.

**Abstract:**

The treatment of acute life-threatening events in patients suffering from chronic pathologies is problematic, as physicians need to consider multisystemic drug effects. Regarding Cerebrolysin, a Sonic Hedgehog signaling pathway amplifier and one of the few approved neurotrophic treatments for stroke patients, concerns of excessive Hedgehog pathway activation that could accelerate NAFLD progression to cirrhosis seem valid. We investigated stroke patients treated with Cerebrolysin that presented elevated levels of aspartate aminotransferase (AST) and/or alanine aminotransferase (ALT). We also investigated the efficiency of Cerebrolysin in reversing the neurogenesis inhibition within the hippocampus in a mouse model of NAFLD by evaluating behavior and histological outcomes. NeuN, BrdU and Iba1 positive signals in the cortex and hippocampus of the animals were also observed. Clinically, Cerebrolysin improved AST levels in a majority of stroke patients with hepatic damage. The same treatment in an experimental setup was able to reverse anxiety-like behavior in MCD mice, reducing their freezing time from 333.61 ± 21.81 s in MCD animals to 229.17 ± 26.28 in treated ones. The use of Cerebrolysin did not improve short-term memory nor rescued cell multiplication in the hippocampus after MCD food intake. Understanding the neuroprotective and neurotrophic effects that drugs have on NAFLD patients can significantly contribute to a suitable therapeutic approach.

## 1. Introduction

Non-alcoholic fatty liver disease (NAFLD) is a global health problem with a prevalence of approximately 25% worldwide [1]. The rising prevalence of NAFLD has been associated with the increased frequency of a plethora of other liver pathologies such as non-alcoholic steatohepatitis (NASH), ‘cryptogenic’ cirrhosis, hepatocellular carcinoma and other end-organ complications of the metabolic syndrome [2]. In order to understand the cellular and molecular pathways involved in this pathology, a series of animal models that mimic NAFLD/NASH are available. These animal models vary from dietary models, such as high-fat diet [3,4], to mutants that have a point mutation of the leptin receptor [5] or to chemically-induced models using CCL4 [6] or thioacetamide [7]. However, one of the most used models of NAFLD/NASH is a methionine/choline-deficient diet (MCD) [8,9,10]. The model offers a mild NAFLD/NASH with male C57BL6 mice developing mostly an inflammatory hepatic pathology with abundant necrosis, lipid peroxidation and ultrastructural injury, which is, histologically, similar to human NASH. The main drawback of the MCD diet model is that mice develop a different metabolic profile than humans [11,12]. Another drawback of the model is that many cellular metabolisms rely on methionine and choline [13,14] and, as such, can interfere with basic molecular findings. For example, methionine is an essential amino acid involved in various processes such as cellular growth and development and protein and enzyme synthesis [13]. Choline is a source of acetylcholine (essential in neuronal signaling), phosphocholine, phosphatidylcholine and sphingomyelin, all important components of the cell membrane [15,16]. It was not surprising that the deprivation of methionine and choline can cause a decrease in survival of neural stem cells (NSCs) and transformation into mature neurons in C57BL/6 mice [17]. This can be problematic, as several groups are investigating cortical influences of systemic pathologies. The reason for this issue is that due to the increase in life expectancy, several acute events will start to affect a larger number of individuals suffering from a chronic disease. As such, physicians will find themselves in front of patients who need treatment with no prior indication of chronic conditions.

With cerebrovascular pathologies reaching an all-time high [18], we wanted to understand how NAFLD patients would be impacted if neuroprotective and neurotrophic drugs were administrated. We focused our efforts on Cerebrolysin, a mixture of low-molecular-weight peptides and amino acids, which can influence the Sonic Hedgehog (Shh) signaling pathway, of great importance in neurogenesis [19,20]. However, preclinical data suggest that excessive Hedgehog (Hh) pathway activation accelerates NAFLD progression to cirrhosis [21,22]. As such, there could be an argument against Cerebrolysin treatment of stroke patients with hepatic injury. In the present study, we aimed to assess variation in liver enzymes in patients receiving Cerebrolysin. As we already showed that Cerebrolysin treatment can increase cortical BrdU signal [12], we also wanted to investigate if Cerebrolysin can prevent the decrease in NSCs in an animal model of NAFLD/NASH thus improving the clinical outcome for these animals when measuring anxiety-like behavior and short-term memory.

## 2. Materials and Methods

### 2.1. Stroke Patients

**Figure 1:**
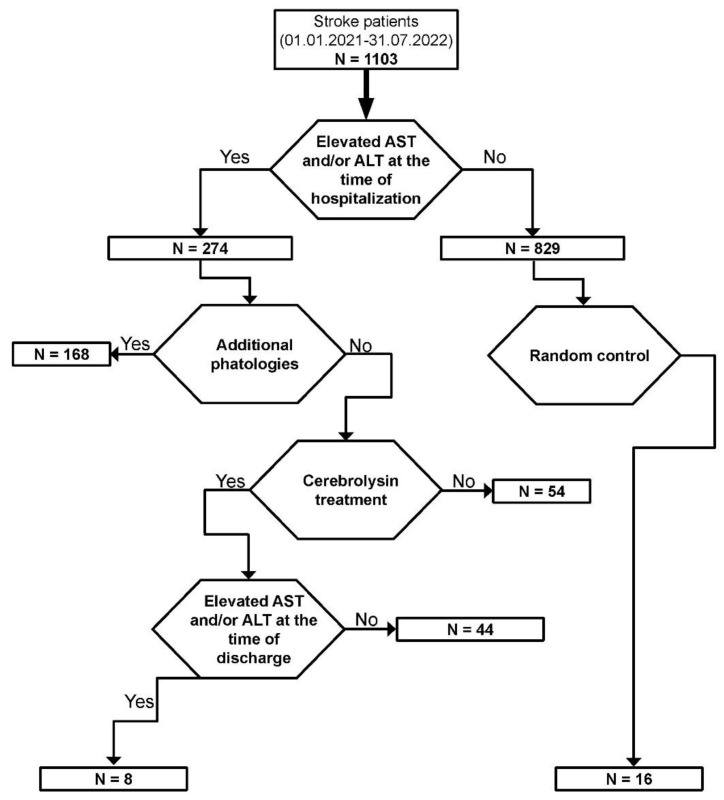
Flow chart of stroke patients selected for the study. From 1103 stroke patients 274 had elevated AST and/or ALT enzymes.

In order to investigate the clinical outcome of Cerebrolysin in patients with liver damage, we retrospectively searched in the database of the Clinical Neuropsychiatry Hospital Craiova (SCNP) (1 January 2021 until 31 July 2022) for ischemic stroke patients, confirmed by CT scan, that also had elevated levels of aspartate aminotransferase (AST) and/or alanine aminotransferase (ALT) at the time of admission (aspartate aminotransferase (AST/GOT) kit, BioSystems, REF 21531). We divided the patients into two groups: treated and untreated with Cerebrolysin. We excluded patients with hemorrhagicor ischemic stroke outside the middle cerebral artery and those suffering from other chronic pathologies that could interfere with the treatment, such as diabetes mellitus, thyroid disorders or chronic renal disease. After the identification, we only selected age-matched patients that had similar changes in the AST and/or ALT levels at admission (Figure 1). The SCNP Craiova Ethical Comity approved the database search (2/02.05.2022).

### 2.2. Experimental Animals

For this study, we used nine-week-old C57BL/6J male (n = 8) and female (n = 8) mice housed individually in a temperature-controlled vivarium (21–23 °C and 60–70% humidity) under a 12 h light/dark cycle, with food and water available ad libitum. All experimental protocols and animal care were conducted according to the guidelines of the Committee for Experimental Animals Wellbeing of the University of Medicine and Pharmacy of Craiova (protocol code 2.13/29.10.2020).

### 2.3. Non-Alcoholic Fatty Liver Disease/Non-Alcoholic Steatohepatitis Induction

Before replacing normal food with pelleted food lacking methionine/choline chloride (MCD) to induce a non-alcoholic, non-viral hepatitis model for non-alcoholic fatty liver disease/non-alcoholic steatohepatitis (MP Biomedicals, Eschwege, Germany) [8,10], mice were allowed a 3-day period to acclimatize to the new laboratory conditions, as they were transferred to a “work room” outside the main colony. After 2 weeks (W2) of MCD food, mice (n = 12) were randomly divided into two groups (6 mice in each group), with an equal number of males and females per group. All MCD mice received intraperitoneal injections daily: the treatment group (MCG + Cy) received 10 mg/kg Cerebrolysin (Ever Pharma, Oberburgau, Austria) and saline treatment was given the control group (MCG). The treatment was always administered in the afternoon during the entirety of the experimental procedures. All MCD animals were fed MCD ad libitum for an additional 4 weeks. The remaining unassigned animals (n = 4) were fed normal food. They also received intraperitoneal saline injections starting with W2.

### 2.4. Clinical Evaluation of Mice and Behavior Testing

Due to the nature of changes induced by the MCD diet, we tested all mice before any MCD intake. In order to investigate anxiety-like behavior and short-term memory alterations we used the novel object recognition test (NOR), recorded and analyzed by an automatic system (EthoVision XT 14, Wageningen, Noldus Technology), as previously described [12]. In short: the animals were placed into an arena with two identical objects, at an equal distance (15 cm from the sidewalls) for a total time of 6 min. After this interval, animals were paced back in their cage for 1 h. The test was repeated, and the mice were allowed to explore the arena for six minutes, where one object was replaced with a novel one. All behavioral variables were recorded and analyzed using the automatic system. At the end of each trial, the arena was cleaned with 75% ethanol in order to remove odors. To quantify memory, we measured the time mice spent exploring each object. We then calculated the preference index (the percentage of the time exploring one identical object within the total time exploring both objects). We also investigated the total freezing time, by adding the time in which the mouse remained immobile.

All animals were inspected daily for any distress and the body weight of each animal was measured weekly. In practice, the mice were tested 3 times: before the start of MCD, 2 weeks after the start of MCD but prior to Cerebrolysin treatment, and after 4 weeks of MCD and Cerebrolysin/serum treatment.

### 2.5. Histopathology and Immunohistochemistry

For tissue harvesting we used a method with the least impact on microglial activation [23]. Anesthetized mice were intracardially perfused with 5 mL saline, followed by 5 mL 4% paraformaldehyde and overnight fixation. Then, some of the tissues were frozen and others were included in paraffin. The liver samples were deparaffined, rehydrated and further stained with hematoxylin and eosin, Periodic Acid Schiff and Masson Trichrome staining. To quantify the hepatic lesion it was used a previous histological scoring for non-alcoholic fatty liver model [24]. For chemical immunohistochemistry, sections were further processed for antigen retrieval in citrate buffer (0.1 M, pH 6) by microwaving them for 20 min at 650 W. After cooling to room temperature, endogenous peroxidase was inhibited with a 1% solution of water peroxide for 30 min, then the unspecific binding sites were blocked in 3% skimmed milk (Biorad, Hercules, CA, USA) for another 30 min. For enzymatic detection, the slides were incubated (18 h) at 4 °C with the primary antibodies (monoclonal Anti-NeuN mouse at a 1:1.000, Millipore Sigma, St. Louis, MO, USA). The next day, the signal was amplified with a species-specific peroxidase-labelled polymer (Nichirei Biosciences, Tokyo, Japan) for 1 h, and visualized with 3,3′-diaminobenzidine (Nichirei Biosciences, Tokyo, Japan). After hematoxylin counterstaining, slides were covered with xylene-based mounting medium (Sigma-Aldrich, St. Louis, MO, USA). Images were collected randomly from the cortex regions of interest (primary somatosensory cortex) and hippocampus, capturing at least three microscopic fields (671.38 × 562.95 μm) for each region. For fluorescence immunohistochemistry, 25 μm thick frozen sections were cut and prepared for immunohistochemistry following standard protocols [25]. To identify macrophages and microglia we used antibodies against Iba1 (1:3000, Wako Chemicals USA Inc., Richmond, VA, USA). Negative controls that omit primary antibodies and positive controls were applied for each case. The positive cells were counted using a 40× magnification in the same cortical region as in the case of chemical immunohistochemistry. For 3 consecutive days after the start of the treatment (W2), all animals received two intraperitoneal injections of 5-bromo-20-deoxyuridine (BrdU, 50 mg/kg; Sigma-Aldrich, St. Louis, MO, USA) (at 8:00 and 20:00). The acquired images were furthered quantified: the area of BrdU and Iba1 signal was quantified using Fiji [26] while the number of NeuN+ cells were manually counted.

### 2.6. Statistical Analysis

For immunohistochemistry results we used multiple comparison one-way ANOVA (Tukey’s multiple comparisons test) after data set passed normality testing. All figures show mean value and standard deviation (SD), and the statistical significance is displayed as follows: * *p* < 0.05, ** *p* < 0.01, *** *p* < 0.001 and **** *p* < 0.0001.

## 3. Results

### 3.1. Cerebrolysin Improves ASL Levels in a Majority of Stroke Patients with Hepatic Damage

**Figure 2:**
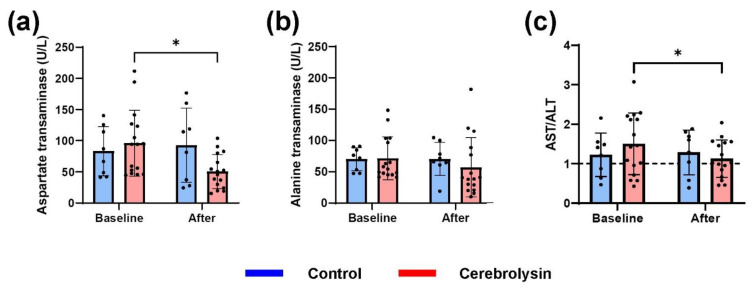
AST and ALT levels in stroke patients with liver damage treated with Cerebrolysin. (**a**) After 10 days of Cerebrolysin, stroke patients had a drop from 96.05 ± 52.75 U/L to 50.53 ± 27.37 U/L in AST levels (*p* = 0.0127) and the stroke patients that did not receive Cerebrolysin had no change in AST levels (from 83.24 ± 39.58 to 92.53 ± 59.56 U/L, *p* > 0.05). (**b**) No changes after the treatment in ALT levels. (**c**) Cerebrolysin lowered the AST/ALT ratio from 1.50 ± 0.78 to 1.12 ± 0.47 (*p* = 0.49). * *p* < 0.05.

Liver enzymes (AST and ALT) were elevated in 24.74% of stroke patients admitted in the investigated period. From all identified patients, a few have also been tested at discharge. We were able to show that patients admitted for stroke treated with Cerebrolysin had lower AST levels after Cerebrolysin treatment (10 mL/day for an average of approximately 10 days) (*p* = 0.0127) (Figure 2a). Age-matched controls had no change in AST levels (83.24 ± 39.58 compared to 92.53 ± 59.56 U/L, *p* > 0.05) (Figure 2a). While Cerebrolysin impacted AST levels, no change could be seen in ALT (Figure 2b). Cerebrolysin lowered the AST/ALT ratio from 1.50 ± 0.78 to 1.12 ± 0.47 (*p* = 0.49) (Figure 2c). Of note, after treatment, two patients had an increase in AST and six in ALT blood levels; all the rest showed an improvement.

### 3.2. Cerebrolysin Reverses Anxiety-like Behavior in MCD Mice

**Figure 3:**
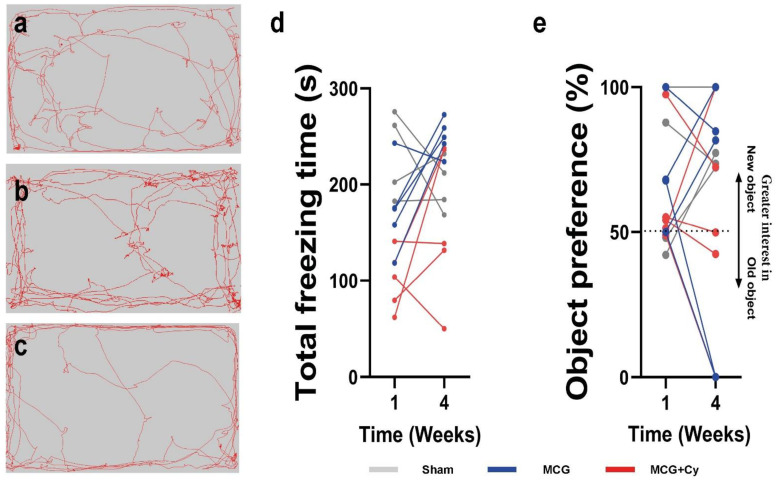
Behavior testing in MCD mice. Novel object recognition test of (**a**)Sham, (**b**) MCD and (**c**) MCD + Cy mice reveals anxiety changes to all mice fed MCD food for 2 weeks. (**d**) When looking at total freezing time, we found the MCD freezing around 272.73 ± 63.67 s, MCD + Cy mice around 296.4 ± 64.77 s, substantially more than the normal-fed animals that maintained a freezing of around 174.17 ± 73.13 s. NOR testing revealed a reversed anxiety-like behavior after 4 weeks of Cerebrolysin, with MCD + Cy mice having a lower freezing time of 229.17 ± 26.28 s compared to 333.61 ± 21.81 s in MCD animals. (**e**) After 4 weeks of Cerebrolysin there was a higher preference average regarding the prefered object in the treated animals of 67.7 ± 28.21% compared with MCD (56.26 ± 26.65%), although not statistically relevant (*p* > 0.05).

Two weeks of MCD food induce anxiety changes to all mice fed MCD (Figure 3a–c). This can be seen as an increase in the MCD group displaying increased freezing behavior. On average, the animals in the MCD group remained immobile around 272.73 ± 63.67 s, while the MCD + Cy mice for 296.4 ± 64.77 s, compared to 174.17 ± 73.13 s recorded for the normal-fed mice (Figure 3d). The administration of Cerebrolysin was able to reverse this anxiety-like behavior after 4 weeks. MCD + Cy mice displayed a lower freezing time of 229.17 ± 26.28 s compared to 333.61 ± 21.81 s in MCD animals (*p* = 0.0023) (Figure 3d). In the NOR test, we measured interest in a new object compared to an existing one. This interest was defined as preference. Although after 4 weeks of treatment there was a higher preference average in the treated animals of 67.7 ± 28.21% compared with MCD (56.26 ± 26.65%), this was not enough to be statistically relevant (*p* > 0.05) (Figure 3e).

### 3.3. Cerebrolysin Cannot Rescue Cell Multiplication in Hippocampus after MCD Food Intake

**Figure 4:**
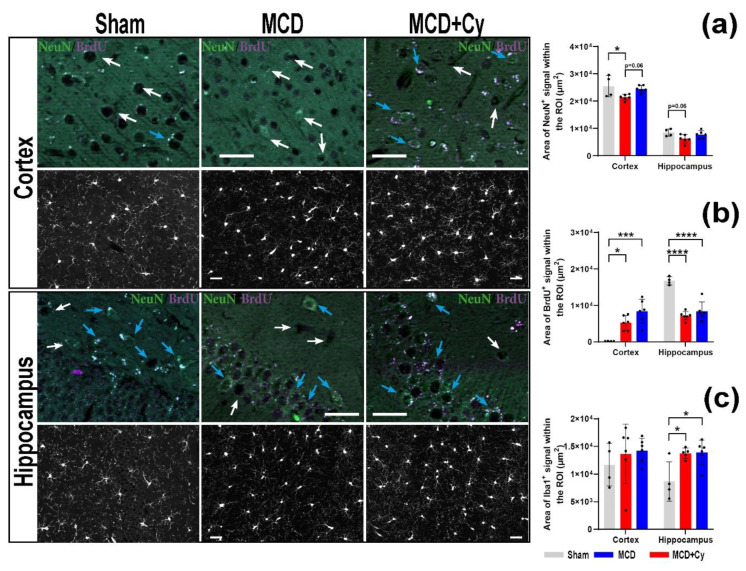
Cellular response to Cerebrolysin in mice fed MCD. (**a**) MCD mice had fewer neurons after 6 weeks of MCD compared to Sham (25,462.32 ± 3946.11 µm^2^ to 21,402.79 ± 1064.86 µm^2^) (*p* = 0.006), while treating mice with Cerebrolysin maintained this value to around 24,476.62 ± 1299.87 µm^2^, comparable to Sham (*p* > 0.05) and different from MCD animals (*p* = 0.021). No impact in the area occupied by NeuN+ cells at the hippocampal level. (**b**) Minimal BrdU signal in the cortex of MCD-fed animals, with no difference between the values found for MCD and MCD + Cy animals (*p* > 0.05). Cerebrolysin treatment failed to increase the area of BrdU signal in the hippocampus of all animals fed MCD food. (**c**) No changes in the area occupied by Iba1+ signal for all tested animals both in the cortex and hippocampus, especially for the hippocampus (13,764.24 ± 919.04 µm^2^ for MCD animals and 13,880.62 ± 2248.69 µm^2^ for MCD + Cy animals, compared to 8693.31 ± 3544.79 µm^2^ in the Sham group). Scale bar 20 µm. The graphs show mean values and SD, * *p* <0.05, *** *p* < 0.001 and **** *p* <0.0001.

We were not able to confirm previously reported findings by showing that Cerebrolysin prevents cortical neuronal loss after MCD food intake (Figure 4a). We found that after 6 weeks of MCD food, mice had a drop in the area of NeuN+ signal from around 25,462.32 ± 3946.11 µm^2^ to 21,402.79 ± 1064.86 µm^2^ (*p* = 0.0307). Treated mice maintained this value to around 24,476.62 ± 1299.87 µm^2^ and compared to Sham (*p* > 0.05) the value did not differ from those recorded for MCD animals (*p* = 0.068) (Figure 4a). At the hippocampal level the treatment had no impact in the area occupied by the NeuN+ cells, with MCD animals having the smallest value of 6234.72 ± 1517.21 µm^2^ compared to Sham (8518.51 ± 1292.36 µm^2^, *p* = 0.065) and MCD + Cy (7739.26 ± 1399.51 µm^2^, *p* > 0.05) animals (Figure 4a).

Minimal BrdU signal was detected in the cortex of MCD-fed animals (Figure 4b), with no difference between the values found for MCD and MCD + Cy animals (*p* = 0.09). The use of Cerebrolysin increased the number of cortical BrdU+ cells compared to Sham (*p* = 0.0004). Even though Cerebrolysin treatment showed encouraging results at the cortical level, it failed to increase the area of BrdU signal in the hippocampus of animals fed MCD food. The MCD food diet generated a drop from around 16,724.17 ± 1 µm^2^ in Sham animals to 7133.04 ± 1169.34 µm^2^ for MCD animals (*p* < 0.0001) and 8345.92 ± 2566.22 µm^2^ for MCD + Cy animals (*p* < 0.0001) (Figure 4b).

No changes in the area occupied by the Iba1+ signal were detected for all tested animals in the cortex (Figure 4c). This is despite the fact that for the cortex the average area of Iba1+ signal was higher in animals fed MCD food. The use of MCD food increased the area of Iba1+ signal in the hippocampus of all animals. The average hippocampal Iba1+ area was around 13,764.24 ± 919.04 µm^2^ for MCD animals. Cerebrolysin was not able to reverse this increase in MCD + Cy animals, having an area of Iba1 around 13,880.62 ± 2248.69 µm^2^ compared to 8693.31 ± 3544.79 µm^2^ in the Sham group (*p* = 0.0131) (Figure 4c).

## 4. Discussion

With treatment options that allow patients suffering from a chronic condition to reach higher life expectancy, in the present clinical practice, hospitalized patients presenting an acute event, such as stroke, frequently have other comorbidities that the physician needs to take in account. Comparable to previous reports [27], in the present study 24.74% of patients admitted for stroke had elevated liver enzymes. The association between stroke and liver damage was investigated in other types of liver damage, for example in alcohol-induced hepatic damage [28], while NAFLD was recently associated with increased risk of stroke [29]. With a plethora of molecular [30,31] and cellular damage [32,33], stroke has devastating consequences. Unfortunately, for a majority of patients medical professionals have limited treatment options, physical recovery being established as the only real option. However, for large cerebral infarctions, the recovery is at best limited, and as such, any improvement is beneficial. In the last decade a large number of neurotrophic factors involved in cortical cell survival, growth and differentiation have been identified and some commercial ones are available. One such example is Cerebrolysin, a solution of low-molecular-weight neuropeptides and free amino acids, previously used in both preclinical and clinical trials [34]. From the initial observation regarding the outcome of patients receiving Cerebrolysin, we are now starting to better understand the molecular machinery that Cerebrolysin can influence. One such example is the Shh pathway, one of the human Hh signaling pathways, that Cerebrolysin can enhance [19]. However, excessive Hh pathway activation in NAFLD seems to determine a progression towards cirrhosis [21,22]. Stroke patients with high liver enzymes receiving Cerebrolysin had a drop from 96.05 ± 52.75 U/L to 50.53 ± 27.37 U/L in AST levels (*p* = 0.0127) after treatment (10 mL/day for an average of approximately 10 days) (Figure 2a). The same patients had an improvement of AST/ALT ratio from 1.50 ± 0.78 at the time of admission to 1.12 ± 0.47 (*p* = 0.49) at discharge (Figure 2c). However, one of the major limitations of this study is that it was a retrospective small sample study, with no long-term measured outcome. The findings do raise some interesting points. One might be that the increase in Shh levels evoked by Cerebrolysin are not sufficient to generate acute additional liver damage in patients, but are sufficient to generate cerebral ones. This might be because Shh had an increase of up to 8-fold in the third degree of steatosis and approximately 7-fold increase in the second degree of steatosis [35], with the highest tested concentration (20 µL/mL) of Cerebrolysin having only a 3-fold change in cerebral Shh [19]. However, for the fourth degree of steatosis the Shh increases in almost unmeasurable [35], making Cerebrolysin, at the very least, problematic to be administered to such patients. Another major problem is that in most of the clinical settings, the recommended use for Cerebrolysin is up to 3 months [36,37]. This exceeds the time window of the present analysis. Thus, extensive long-term investigations regarding the use of Cerebrolysin in NAFLD patients should be considered. One major aspect might be that the Shh pathway has a moderate profibrogenic potency [38], potentially proving not to have a major impact in less severe stages of NAFLD.

Some studies show that depression has a higher frequency in NAFLD patients [1,39]. We could confirm by behavior testing that two weeks of MCD food can induce anxiety-like behavior changes in all mice fed MCD food (Figure 3a,c). Interestingly, 4 weeks of Cerebrolysin treatment could reverse these behaviors, as MCD + Cy mice displayed freezing behavior for 229,17 ± 26,28 s, far less that MCD animals that increased their freezing behavior to 333.61 ± 21.81 s (*p* = 0.0023) (Figure 3d). This might prove of importance, as chronic liver injury could induce depressive-like behavior in mice [40] and chronic stress status may be an important risk factor of NAFLD. As such, with the proper Shh monitoring, Cerebrolysin could overall be beneficial for patients with liver damage, even in a chronic administration. Although in other experimental settings such as post-traumatic stress disorder [41] or sleep deprivation [42] the treatment was not able to prevent short-term memory loss seen in animals fed MCD food (Figure 3e), there is a higher preference average in the treated animals of 67.7 ± 28.21% compared with MCD (56.26 ± 26.65%). This could be due to the small sample size of animals investigated. However, if the effect is minimal in the experimental setting it is likely that, even if present, the effect will have little overall impact in the clinical practice.

There are several studies investigating the effects of various diets on adult hippocampal neurogenesis (AHN) starting from high-fat diets [43] to those deficient in thiamine [28,44], zinc [45], folate [46], omega-3 fatty acids [47], flavonoids [48] and curcumin [49], with different effects of AHN. One interesting such study looked at AHN in the MCD diet in C57BL/6J male mice, showing that 4 weeks of MCD food induced a decrease in the proliferation, differentiation and survival of NSCs into mature neurons [17]. We were able to confirm previous findings that Cerebrolysin generated a moderate increase in the area of BrdU signal in the cortex [12]; however, it was not able to elicit similar result at the hippocampal level, confirming reports that MCD food inhibits AHN [47]. This implies that any research conducted on the AHN in MCD animals is prone to have at least questionable results.

With Cerebrolysin treatment previously shown to have anti-inflammatory effects both on cell cultures [50,51] and in animal use [52], the lack of any anti-inflammatory effect observed was a surprise. As such, the increase in the area of Iba1+ signal at the hippocampal level might show that its anti-inflammatory effects are most likely a consequence of its neuroprotection rather than a primary outcome.

## 5. Conclusions

Given the prevalence of non-alcoholic fatty liver disease, there is a high chance that some of these patients can develop acute life-threatening events such as stroke. With limited treatment options for most stroke patients, the use of Cerebrolysin does not seem to increase liver damage for patients also suffering from NAFLD prior to discharge. Furthermore, the present model of mouse NAFLD was able to protect against cortical neuronal loss, but had no impact in the hippocampal inflammation seen in MCD mice. Further steps are needed to understand the impact of drugs used in an acute setting on the long-term course of the underlining chronic disease that patients might suffer. In this case, an understanding of the neuroprotective and neurotrophic effects that drugs have on NAFLD patients contributes significantly to a suitable therapeutic approach.

## Data Availability

Not applicable.

## References

[1] Cho I.Y., Chang Y., Sung E., Kang J.H., Wild S.H., Byrne C.D., Shin H., Ryu S. (2021). Depression and increased risk of non-alcoholic fatty liver disease in individuals with obesity. Epidemiol. Psychiatr. Sci..

[2] Youssef N.A., Abdelmalek M.F., Binks M., Guy C.D., Omenetti A., Smith A.D., Diehl A.M., Suzuki A. (2013). Associations of depression, anxiety and antidepressants with histological severity of nonalcoholic fatty liver disease. Liver Int..

[3] Zamin I., Mattos A.A., Mattos A.Z., Migon E., Soares E., Perry M.L. (2009). Model of experimental nonalcoholic steatohepatitis from use of methionine and choline deficient diet. Arq. Gastroenterol..

[4] Lieber C.S., Leo M.A., Mak K.M., Xu Y., Cao Q., Ren C., Ponomarenko A., DeCarli L.M. (2004). Model of nonalcoholic steatohepatitis. Am. J. Clin. Nutr..

[5] Trak-Smayra V., Paradis V., Massart J., Nasser S., Jebara V., Fromenty B. (2011). Pathology of the liver in obese and diabetic ob/ob and db/db mice fed a standard or high-calorie diet. Int. J. Exp. Pathol..

[6] Tsuchida T., Lee Y.A., Fujiwara N., Ybanez M., Allen B., Martins S., Fiel M.I., Goossens N., Chou H.I., Hoshida Y. (2018). A simple diet- and chemical-induced murine NASH model with rapid progression of steatohepatitis, fibrosis and liver cancer. J. Hepatol..

[7] Fujii M., Shibazaki Y., Wakamatsu K., Honda Y., Kawauchi Y., Suzuki K., Arumugam S., Watanabe K., Ichida T., Asakura H. (2013). A murine model for non-alcoholic steatohepatitis showing evidence of association between diabetes and hepatocellular carcinoma. Med. Mol. Morphol..

[8] Itagaki H., Shimizu K., Morikawa S., Ogawa K., Ezaki T. (2013). Morphological and functional characterization of non-alcoholic fatty liver disease induced by a methionine-choline-deficient diet in C57BL/6 mice. Int. J. Clin. Exp. Pathol..

[9] Rizki G., Arnaboldi L., Gabrielli B., Yan J., Lee G.S., Ng R.K., Turner S.M., Badger T.M., Pitas R.E., Maher J.J. (2006). Mice fed a lipogenic methionine-choline-deficient diet develop hypermetabolism coincident with hepatic suppression of SCD-1. J. Lipid Res..

[10] Lyman R.L., Giotas C., Medwadowski B., Miljanich P. (1975). Effect of low methionine, choline deficient diets upon major unsaturated phosphatidyl choline fractions of rat liver and plasma. Lipids.

[11] Leclercq I.A., Da Silva Morais A., Schroyen B., Van Hul N., Geerts A. (2007). Insulin resistance in hepatocytes and sinusoidal liver cells: Mechanisms and consequences. J. Hepatol..

[12] Morega S., Cătălin B., Simionescu C.E., Sapalidis K., Rogoveanu I. (2021). Cerebrolysin Prevents Brain Injury in a Mouse Model of Liver Damage. Brain Sci..

[13] Young S.N., Shalchi M. (2005). The effect of methionine and S-adenosylmethionine on S-adenosylmethionine levels in the rat brain. J. Psychiatry Neurosci..

[14] Vučević D.B., Cerović I.B., Mladenović D.R., Vesković M.N., Stevanović I., Jorgačević B.Z., Ješić Vukićević R., Radosavljević T.S. (2016). Methionine-choline deprivation alters liver and brain acetylcholinesterase activity in C57BL6 mice. Gen. Physiol. Biophys..

[15] Blusztajn J.K., Liscovitch M., Richardson U.I. (1987). Synthesis of acetylcholine from choline derived from phosphatidylcholine in a human neuronal cell line. Proc. Natl. Acad. Sci. USA.

[16] Ueland P.M. (2011). Choline and betaine in health and disease. J. Inherit. Metab. Dis..

[17] Kim J.W., Hahn K.R., Yoo D.Y., Jung H.Y., Hwang I.K., Seong J.K., Yoon Y.S. (2019). Methionine-Choline Deprivation Impairs Adult Hippocampal Neurogenesis in C57BL/6 Mice. J. Med. Food.

[18] Capuano E., Marchese F., Capuano R., Piramide N., Palumbo R., Simonis V., Iannone A.G., Pironti S., Capuano V. (2021). The Burden of Cardio-Cerebrovascular Risk Factors: Differences Between Individual Risk and Population Risk. High Blood Press. Cardiovasc. Prev..

[19] Zhang L., Chopp M., Meier D.H., Winter S., Wang L., Szalad A., Lu M., Wei M., Cui Y., Zhang Z.G. (2013). Sonic hedgehog signaling pathway mediates cerebrolysin-improved neurological function after stroke. Stroke.

[20] Ziganshina L.E., Abakumova T., Hoyle C.H. (2020). Cerebrolysin for acute ischaemic stroke. Cochrane Database Syst. Rev..

[21] Verdelho Machado M., Diehl A.M. (2018). The hedgehog pathway in nonalcoholic fatty liver disease. Crit. Rev. Biochem. Mol. Biol..

[22] Guy C.D., Suzuki A., Zdanowicz M., Abdelmalek M.F., Burchette J., Unalp A., Diehl A.M. (2012). Hedgehog pathway activation parallels histologic severity of injury and fibrosis in human nonalcoholic fatty liver disease. Hepatology.

[23] Cătălin B., Stopper L., Bălşeanu T.A., Scheller A. (2017). The in situ morphology of microglia is highly sensitive to the mode of tissue fixation. J. Chem. Neuroanat..

[24] Kleiner D.E., Brunt E.M., Van Natta M., Behling C., Contos M.J., Cummings O.W., Ferrell L.D., Liu Y.C., Torbenson M.S., Unalp-Arida A. (2005). Design and validation of a histological scoring system for nonalcoholic fatty liver disease. Hepatology.

[25] Surugiu R., Catalin B., Dumbrava D., Gresita A., Olaru D.G., Hermann D.M., Popa-Wagner A. (2019). Intracortical Administration of the Complement C3 Receptor Antagonist Trifluoroacetate Modulates Microglia Reaction after Brain Injury. Neural Plast..

[26] Schindelin J., Arganda-Carreras I., Frise E., Kaynig V., Longair M., Pietzsch T., Preibisch S., Rueden C., Saalfeld S., Schmid B. (2012). Fiji: An open-source platform for biological-image analysis. Nat. Methods.

[27] Cholongitas E., Pavlopoulou I., Papatheodoridi M., Markakis G.E., Bouras E., Haidich A.B., Papatheodoridis G. (2021). Epidemiology of nonalcoholic fatty liver disease in Europe: A systematic review and meta-analysis. Ann. Gastroenterol..

[28] Yilmaz I., Demiryilmaz I., Turan M.I., Çetin N., Gul M.A., Süleyman H. (2015). The effects of thiamine and thiamine pyrophosphate on alcohol-induced hepatic damage biomarkers in rats. Eur. Rev. Med. Pharmacol. Sci..

[29] Wang M., Zhou B.-G., Zhang Y., Ren X.-F., Li L., Li B., Ai Y.-W. (2022). Association Between Non-alcoholic Fatty Liver Disease and Risk of Stroke: A Systematic Review and Meta-Analysis. Front. Cardiovasc. Med..

[30] Cetin N., Dasdelen D., Mogulkoc R., Menevse E., Baltaci A.K. (2022). Role of exogenous putrescine in the status of energy, DNA damage, inflammation, and spermidine/spermine-n(1)-acetyltransferase in brain ischemia-reperfusion in rats. Iran. J. Basic Med. Sci..

[31] Balseanu A.T., Buga A.M., Catalin B., Wagner D.C., Boltze J., Zagrean A.M., Reymann K., Schaebitz W., Popa-Wagner A. (2014). Multimodal Approaches for Regenerative Stroke Therapies: Combination of Granulocyte Colony-Stimulating Factor with Bone Marrow Mesenchymal Stem Cells is Not Superior to G-CSF Alone. Front. Aging Neurosci..

[32] Pirici I., Balsanu T.A., Bogdan C., Margaritescu C., Divan T., Vitalie V., Mogoanta L., Pirici D., Carare R.O., Muresanu D.F. (2017). Inhibition of Aquaporin-4 Improves the Outcome of Ischaemic Stroke and Modulates Brain Paravascular Drainage Pathways. Int. J. Mol. Sci..

[33] Popescu E.S., Pirici I., Ciurea R.N., Balseanu T.-A., Catalin B., Margaritescu C., Mogoanta L., Hostiuc S., Pirici D. (2017). Three-dimensional organ scanning reveals brain edema reduction in a rat model of stroke treated with an aquaporin 4 inhibitor. Rom. J. Morphol. Embryol..

[34] Heiss W.D., Brainin M., Bornstein N.M., Tuomilehto J., Hong Z. (2012). Cerebrolysin in patients with acute ischemic stroke in Asia: Results of a double-blind, placebo-controlled randomized trial. Stroke.

[35] Estep M., Mehta R., Bratthauer G., Alaparthi L., Monge F., Ali S., Abdelatif D., Younoszai Z., Stepanova M., Goodman Z.D. (2019). Hepatic sonic hedgehog protein expression measured by computer assisted morphometry significantly correlates with features of non-alcoholic steatohepatitis. BMC Gastroenterol..

[36] Muresanu D.F., Heiss W.D., Hoemberg V., Bajenaru O., Popescu C.D., Vester J.C., Rahlfs V.W., Doppler E., Meier D., Moessler H. (2016). Cerebrolysin and Recovery After Stroke (CARS): A Randomized, Placebo-Controlled, Double-Blind, Multicenter Trial. Stroke.

[37] Mureșanu D.F., Popa L.L., Chira D., Dăbală V., Hapca E., Vlad I., Văcăraș V., Popescu B.O., Cherecheș R., Strilciuc Ș. (2022). Role and Impact of Cerebrolysin for Ischemic Stroke Care. J. Clin. Med..

[38] Schwabe R.F., Tabas I., Pajvani U.B. (2020). Mechanisms of Fibrosis Development in Nonalcoholic Steatohepatitis. Gastroenterology.

[39] Xiao J., Lim L.K.E., Ng C.H., Tan D.J.H., Lim W.H., Ho C.S.H., Tan E.X.X., Sanyal A.J., Muthiah M.D. (2021). Is Fatty Liver Associated with Depression? A Meta-Analysis and Systematic Review on the Prevalence, Risk Factors, and Outcomes of Depression and Non-alcoholic Fatty Liver Disease. Front. Med..

[40] Zhu L.L., Shen J.D., Wang B.Y., Lu S.F., Ming B., Xu E.P., Li Y.C., Fang X.Y. (2020). Depressive-like behaviors are induced by chronic liver injury in male and female mice. Neurosci. Lett..

[41] Alzoubi K.H., Al-Ibbini A.M., Nuseir K.Q. (2018). Prevention of memory impairment induced by post-traumatic stress disorder by cerebrolysin. Psychiatry Res..

[42] Alzoubi K.H., Al-Jamal F.F., Mahasneh A.F. (2020). Cerebrolysin prevents sleep deprivation induced memory impairment and oxidative stress. Physiol. Behav..

[43] Hwang I.K., Kim I.Y., Kim D.W., Yoo K.Y., Kim Y.N., Yi S.S., Won M.H., Lee I.S., Yoon Y.S., Seong J.K. (2008). Strain-specific differences in cell proliferation and differentiation in the dentate gyrus of C57BL/6N and C3H/HeN mice fed a high fat diet. Brain Res..

[44] Zhao N., Zhong C., Wang Y., Zhao Y., Gong N., Zhou G., Xu T., Hong Z. (2008). Impaired hippocampal neurogenesis is involved in cognitive dysfunction induced by thiamine deficiency at early pre-pathological lesion stage. Neurobiol. Dis..

[45] Corniola R.S., Tassabehji N.M., Hare J., Sharma G., Levenson C.W. (2008). Zinc deficiency impairs neuronal precursor cell proliferation and induces apoptosis via p53-mediated mechanisms. Brain Res..

[46] Kronenberg G., Harms C., Sobol R.W., Cardozo-Pelaez F., Linhart H., Winter B., Balkaya M., Gertz K., Gay S.B., Cox D. (2008). Folate deficiency induces neurodegeneration and brain dysfunction in mice lacking uracil DNA glycosylase. J. Neurosci..

[47] Kawakita E., Hashimoto M., Shido O. (2006). Docosahexaenoic acid promotes neurogenesis in vitro and in vivo. Neuroscience.

[48] An L., Zhang Y.Z., Liu X.M., Yu N.J., Chen H.X., Zhao N., Yuan L., Li Y.F. (2011). Total flavonoids extracted from xiaobuxin-tang on the hyperactivity of hypothalamic-pituitary-adrenal axis in chronically stressed rats. Evid. Based Complement. Altern. Med..

[49] Kim S.J., Son T.G., Park H.R., Park M., Kim M.S., Kim H.S., Chung H.Y., Mattson M.P., Lee J. (2008). Curcumin stimulates proliferation of embryonic neural progenitor cells and neurogenesis in the adult hippocampus. J. Biol. Chem..

[50] Alvarez X.A., Lombardi V.R., Fernández-Novoa L., García M., Sampedro C., Cagiao A., Cacabelos R., Windisch M. (2000). Cerebrolysin reduces microglial activation in vivo and in vitro: A potential mechanism of neuroprotection. J. Neural Transm. Suppl..

[51] Lombardi V.R., Windisch M., García M., Cacabelos R. (1999). Effects of Cerebrolysin on in vitro primary microglial and astrocyte rat cell cultures. Methods Find Exp. Clin. Pharmacol..

[52] Guan X., Wang Y., Kai G., Zhao S., Huang T., Li Y., Xu Y., Zhang L., Pang T. (2019). Cerebrolysin Ameliorates Focal Cerebral Ischemia Injury Through Neuroinflammatory Inhibition via CREB/PGC-1α Pathway. Front. Pharmacol..

